# Women Caring for Husbands Living with Parkinson’s Disease: A Phenomenological Study Protocol

**DOI:** 10.3390/jpm12050659

**Published:** 2022-04-20

**Authors:** Dawn R. White, Patrick Albert Palmieri

**Affiliations:** 1 College of Graduate Health Studies, A.T. Still University, 800 W. Jefferson Street, Kirksville, MO 63501, USA; dawn.white.phd@gmail.com; 2 Department of Health Sciences, Centura College, 932 Ventures Way, Chesapeake, VA 23320, USA; 3 South American Center for Qualitative Research, Universidad Norbert Wiener, Avenida Arequipa 440, Lima 15046, Peru; 4 EBHC South America: A JBI Affiliated Group, Calle Cartavio 406, Suite 402, Lima 15023, Peru; 5 Center for Global Nursing, Texas Woman’s University, 6700 Fannin St., Houston, TX 77030, USA

**Keywords:** Parkinson’s disease, caregiving, caregiver burden, spouses, fatigue, qualitative research

## Abstract

Parkinson’s disease (PD) is an emerging pandemic caused by aging, longevity, and industrialization. Most people diagnosed with PD initially experience mild symptoms, but over time the symptoms become debilitating. Given their intensive care requirement, most married people living with PD receive care from their spouses; most are female caregivers. Because caregiving is hard work with long hours, caregivers experience stress, fatigue, and depression, often leading to exhaustion and burnout. The purpose of this descriptive phenomenological study is to understand the lived experience of women caregivers of husbands living with PD. As part of this study protocol, women caring at home for their husbands diagnosed with PD will be purposely recruited from the Colorado Parkinson Foundation. Semi-structured interviews will be conducted by Zoom© until data saturation is achieved. Colaizzi’s seven-step process will be used to analyze the data in Atlas.ti. Strategies have been incorporated into the study protocol to maximize trustworthiness and to insure methodological rigor. The study will be reported using recommendations from the Standards for Reporting Qualitative Research and the Consolidated Criteria for Reporting Qualitative Research. Findings from this study may guide intervention development to improve the caregiving experience and to inform clinical practice guidelines for health care professionals.

## 1. Introduction

Parkinson’s disease (PD) was recognized in 1817 when Dr. James Parkinson investigated six people in England who had similar symptoms but without a diagnosis [1]. By 1855, 22 people were diagnosed with the disease [2]. Dr. Parkinson described the disease as shaking palsy [1] because the people he observed had tremors, cognitive dysfunction, and rigidity that impacted their quality of life [3]. Later, Dr. Charcot, a French neurologist, reevaluated shaking palsy and formally renamed it Parkinson’s disease [4]. Although PD is a neurodegenerative disorder without a known cure, treatment strategies can help manage individual symptoms [5]. Importantly, the prevalence of PD increases with an aging population and global industrialization. Furthermore, people living with PD are 68% more likely to be male [6], typically because of their significantly greater risk from previous chemical exposures [7], such as pesticides and herbicides.

With disease prevalence increasing because of aging and industrialization, PD is a pandemic [8]. From 1990 to 2015, the number of people diagnosed with PD grew by 118% worldwide, almost doubling in frequency [9]. By 2040, the disease is expected to double again, impacting more than 12 million people worldwide [10]. Parkinson’s disease is the second most common neurodegenerative disease behind Alzheimer’s disease, affecting 2% to 3% of people aged over 65 years [11,12]. In the United States, at least 1 million people are diagnosed with PD, resulting in an estimated economic burden greater than $52 billion [13,14]. By 2037, 1.6 million Americans will be diagnosed with PD, and the projected economic impact will be more than USD 79 billion [15].

## 2. Background

People living with PD initially experience mild symptoms, and over time the symptoms become more severe. In the early stages of PD, patients notice mild motor and nonmotor symptoms, such as tremors, brain fog, and increased mental health troubles such as depression and anxiety [16,17]. They also experience more significant comorbidities, such as gastrointestinal and cardiac disorders [15]. As the illness progresses, people living with PD become more symptomatic and less able to care for themselves. The Hoehn and Yahr scale [18] is widely used to grade PD severity [19]. The system is rated in five stages (Table 1). Stages 1 and 2 represent people with minimal disability who are still able to live their lives with limited dependence on caregivers, and the remaining three stages translate into the progressive need for caregivers to manage activities of daily living (ADLs) [20].

After many years of disease progression, people living with PD begin to miss work, require more medical care, and eventually require assistance with most activities [12]. The disease eventually results in small tasks learned as a child, such as picking up food with a fork requiring caregiver assistance. A caregiver is a person who assists someone living with a chronic condition or physical disability, usually without compensation, external to a professional, employment, or other formal framework [21]. As the disease advances, the caregiver becomes progressively responsible for the ADLs [22]. Caregivers are essential to reduce morbidity and mortality and to increase the quality of life of people living with PD [23]. In addition, caregivers reduce the direct medical costs for nursing home care by nearly USD 100,000 per year [24]. Given the disease complexity, care burden, and medical costs, most married people living with PD receive care from their spouses [25]; most of these caregivers are female [26].

**Table 1:** Hoehn and Yahr Stages.

Stages	Parkinson’s Disease (PD) Symptoms	Implications for Activities of Daily Living (ADLs)	Caregiver Responsibilities for People Living with PD
1	Unilateral symptoms but can also involve the neck and spine, usually no noticeable tremor.	No symptoms different from aging, no observable disability, no observable impairment, usually unaware of disease.	Life as normal, diagnosis is usually not made at this stage, disease mistaken for process of aging.
2	Bilateral symptoms but no noticeable impairment of postural reflexes, mild tremor could be present.	Mild symptoms, slower movement and minor balance issues, some mild cognitive confusion such as “brain fog”.	Some assistance might be required for balance issues, when tremor is present, diagnosis is more likely.
3	Bilateral symptoms, mild to moderate impaired postural reflexes, physically independent, tremor.	Mild to moderate symptoms, postural instability, freezing with movement; noticeable tremors; cognitive decline.	Minimal assistance with movement for balance issues, increased risk for falls, ADLs are usually independent.
4	Severe disability, but still able to walk or stand unassisted.	Severe symptoms, difficult to stand/walk without assistance, not able to live independently.	Assistance for movement, usually with a walker, some ADL assistance.
5	Wheelchair required or bedridden without assistance.	Severe symptoms, physically disabled, hallucinations and/or delusions possible.	Complete care, confined to bed or wheelchair; most ADLs require assistance.

References: Goetz et al. [19], Hoehn & Yahr [18], Modestino et al. [20], Parkinson’s Foundation [27].

Typically, a caregiver is a person who thinks of others first and does what is needed for everyone to succeed [28]. In the context of a person living with PD, the caregiver is involved in all aspects of satisfying their needs to make life easier; however, caregiving can have a negative impact on the quality of life of the primary caregiver [17], usually the female spouse [29]. In addition to their responsibilities, caregivers go through a litany of emotions, including frustration, resentment, anger, sadness, and an overall fear of what will happen in their future [30]. This work is quite difficult, and caregivers describe their sense of physical strain and emotional distress, complicated by financial hardship associated with the economic burden of the disease [23]. Each person living with PD has an individually distinct experience; thus, spouses are rarely able to anticipate the challenges of caregiving [31].

Because PD alters the concept of being a wife, feelings of disconnection become a shared experience for caregivers. After the disease diagnosis, these women describe their experience as feeling “disharmony, disequilibrium, disability, and disease which incorporates a loss of the familiar world” [32]. Caregiving places a wife in a familiar but more complex role since their spouse requires more effort to sustain physical wellness. For instance, they often provide physical assistance essential for mobility and movement. Because 65% of caregivers are female spouses [33] aged over 65 years who have their own limitations, the work commitment is physically demanding [34,35].

Managing the overwhelming feelings of stress and anxiety requires strategies. For example, outside activities can be pursued to break up the monotony of living with a spouse with a neurodegenerative disease. The spousal caregiver needs to continue with outside activities that sustain their personal requirements as human beings [36]. By distracting their psyche from daily caregiving activities, these women can rest their bodies and relax their minds. However, when the wives attend functions or participate in group activities with their husbands, they remain in the caregiver role rather than the role of a wife enjoying time outside the home [28]. Because wife caregivers are dedicated to helping their husbands feel comfortable in social settings by facilitating their mobility, they experience a diminished personal identity outside the home.

## 3. Caregiver Burden and Benefit

Caregiver burden is a common outcome when caring for people living with a chronic disorder, such as PD [6]. When caregivers perceive caring as a burden, they report discomfort while providing care as limitations in emotional, financial, social, physical, and spiritual functions [12]. Caregivers also experience internal conflict when deciding whether to care for their loved ones or for themselves. They can also experience stigma by association related to the symptoms of the person living with PD [37]. In most circumstances, caregivers view their choice as dichotomous because they feel strongly about putting their needs aside for a later time that never comes [12].

Since caring usually continues for years, this phenomenon is referred to as a caregiving career [38], where the burden felt by the caregiver steadily increases in severity as the PD symptoms progress. If the burden is not mitigated, the caregiver eventually burns out or experiences complete exhaustion [29]. Caregiver burden can be measured with surveys and questionnaires, which help identify the presence of the problem. However, caregivers also need to share their lived experiences to appreciate the etiology of problems and to develop solutions that minimize the caregiving career impact on their personal lives [25].

Caregivers of people living with PD usually work more than 50 h per week in their caregiver role [26]. A common theme in the caregiver literature is physical and mental fatigue. As described by Kang et al. [35], fatigue explains the constellation of negative psychological conditions resulting from caregiver burden, such as stress, anxiety, and depression. Typically, fatigue can be relieved with rest and relaxation. However, caregivers usually continue to work when faced with the hourly demands of caring for their husbands. If fatigue is experienced for longer durations of more than six consecutive months, caregivers often experience depressive symptoms [16] and a reduced quality of life [35], which eventually results in exhaustion [39]. For caregivers of people living with PD, exhaustion is described as tense muscles, severe stress, and emotional drain, which usually occurs immediately before caregiver burnout [36]. Therefore, understanding caregiving through the lived experiences of caregivers is the best way to develop strategies to prevent caregiver burden and eventual burnout.

Although the literature mostly focuses on the negative aspects of caring for someone living with a degenerative neurocognitive disease, such as PD, some caregivers experience positive benefits. Caregivers report they are more compassionate and learn to be more patient [33]. Because helping someone is satisfying, most caregivers have reported caregiving as a positive experience [40] that contributes to personal growth and a feeling of a life purpose. In a small observational study, even when caregivers reported feeling stressed about caregiving, they were also positive about their quality of life [41]. However, quality of life has been reported to significantly decrease with longer durations of PD [42]. The most substantial caregiver benefit has been reported in couples where the spouse recognizes the caregiver for their dedication in helping with the disease and staying in the relationship [34].

## 4. Conceptual Framework for Caregiving

Theory supports research by explaining why things happen or why people view life in a certain way. Theories organized into conceptual frameworks are important for understanding caregiving [43]. Using a conceptual framework, researchers create a “map of how all of the literature works together in a particular way” [44]. In the current phenomenological study, the phenomenon being studied is women caregivers who are caring for husbands living with PD. The study is guided by the interaction of three theories to conceptualize their lived experiences of caregiving.

**Figure 1:**
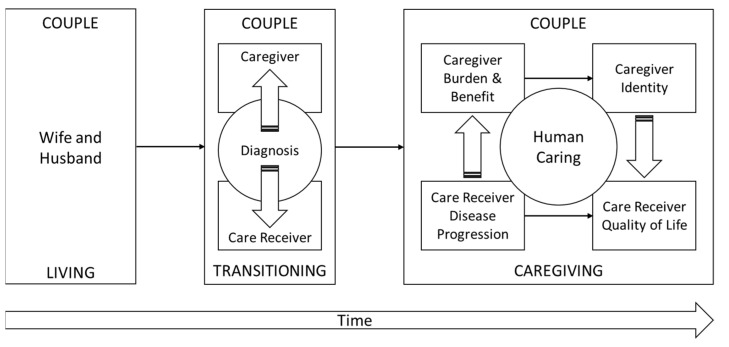
Conceptual Framework for Caregiver Transition in Parkinson’s Disease.

Caregiver identity theory [45,46], self-determination theory [47], and the theory of human caring [48,49,50,51] may help explain caregiver burdens, such as fatigue, sadness, anger, and lack of identity, and caregiver benefits, such as spirituality, finding inner strength, and true commitment to the spouse they love. As such, these three theories inform caregiving as a phenomenon where a wife transitions to caregiver because of a husband’s PD diagnosis. To better understand caregiving within the context of these theories, more research is necessary to understand the lived experience of caregivers, including the associated benefits and burdens. As shown in Figure 1, the interaction of these theories is conceptualized for caregiving as the model.

### 4.1. Caregiver Identity Theory

Caregiver identity theory explains why wives care for their husbands living with neurodegenerative diseases [38]. Initially developed by Montgomery and Kosloski [45], this theory explains how the role of each caregiver is shaped by culture and family dynamics. As such, the wife will only recognize the need to use external services when there is a discrepancy in personal perspective about her ability to engage in caring activities, the perspective about the relationship with the care receiver changes, or there is a major change in the caregiving context [46].

As part of the process of becoming a caregiver, the person experiences changes in behaviors and a shift in role identity [52]; in the case of the spousal caregiver, the role transitions from an equal partner in an intimate familial relationship to a more servant-like relationship with differential power [30]. The process results in a gradual transition from the wife managing the entire house, including the finances, to progressively assuming responsibility for the ADLs of her husband. The role is at variance with the social norms of a mature female spouse. Consequently, the caregiver experiences an internal conflict that forces them to choose between fulfilling their own needs or satisfying the needs of their husband living with PD [12], resulting in isolation, anxiety, and role strain as the dyadic relationship between the caregiver and incapacitated person deteriorates [32]. Managing this conflict results in the caregiver altering their identity, ultimately losing a part of who they are or once were [38].

### 4.2. Self-Determination Theory

Caregiving is a long-term commitment. Assuming this role causes increased stress and a progressive loss of self-identity; however, caregivers may be able to maintain an identity equilibrium through self-motivation. More specifically, self-determination theory explains how motivation can result from caregiving. People with high motivation and willingness to identify autonomy, competence, and relatedness have a more positive mental health status [47]. If caregivers are unable to achieve a sufficient level of autonomy, competence, and relatedness, they often experience mental health problems and their well-being deteriorates [53]. For this reason, there are negative impacts and positive values associated with caregiving [54,55]. Since PD is a progressive neurodegenerative disorder, the wife assumes total care responsibility for her husband in a gradual transition rather than a sudden process. As such, self-determination theory is relevant for understanding the protective mechanisms and coping strategies women utilize to strengthen their self-identity and to maintain equilibrium in their caregiving relationship.

### 4.3. Theory of Human Caring

The characteristics that define human caring [50] are necessary to understand the context of caring relationships [48]. Although the theory of human caring originally focused on explaining how nurses holistically care for people living with diseases [49], the theory is now broadly applied to caring in the context of health, wellness, and illness [56]. As a science [57], human caring is a measured phenomenon [58] characterized by the ten caritas processes, caring relationships, and caring moments [51]. People living with serious diseases require a caregiving approach infused with kindness, faith, hope, trust, spirituality, and awareness of sensitivity [59]. As an intentional human responsibility, caring is foundational for assuming the caregiving responsibility for people living with PD, and the ethic of love contextualizes the caring relationship [60]. Therefore, defining human caring in the context of PD may be an important pathway to better understand the relationship between caregiver benefit and burden. Admirable caregiver intentions resulting from the spousal relationship may manifest as caregiver benefit derived from human caring. Similar to the theory of human caring, women caregivers of husbands living with PD use coping skills to continue their caring role [61], including taking one day at a time, praying and relaxing.

## 5. Purpose of the Study

Because PD is a progressive neurological disorder that alters motor and nonmotor function, it can cause tremors, slow movements, stiff muscles, an unsteady walk, and balance and coordination problems [16,17]. With disease progression, deceased cognitive function is common, including forgetfulness and trouble with concentration. As the demanding job of caring for a person with PD progresses, the wife assumes more responsibility, transitioning from a spouse to a caregiver [62]. Unfortunately, physicians have limited knowledge about how to help caregivers [63]. As a result, wives experience additional pressures and fatigue, eventually becoming a faceless caregiver instead of a spouse visiting the physician office’s with their husband [64]. This situation has recently been described as caregivers becoming “invisible” patients [65].

The purpose of the current descriptive phenomenological study is to understand the lived experience of wives caring at home for their husbands living with PD. Although previous studies have reported quantitative findings about spousal caregiving, few studies provide insights about the lived experiences of wife caregivers. Furthermore, the daily activities of caregivers are poorly understood, including how they negotiate daily activities, manage medications, and assist with physical therapy [66]. With improved understanding of caregiving in the home setting, caregivers can be better supported to continue their work into the late stages of the disease [67].

Based on constructs identified in the literature specific to caregivers and PD, our research question about the lived experience of wives caring for husbands living with PD was developed. To answer the research question, the study has the following four aims: (1) explore caregiving in the context of married couples impacted by PD, (2) identify the burdens associated with caregiving in the context of PD, (3) identify the benefits derived from caregiving in the context of PD, and (4) solicit perspectives about how to improve the lived experience of wives caring for their husbands living with PD. The current article describes the study protocol to address our research question and study aims.

## 6. Methods

People learn from their own experiences and those of other people; they then use those experiences to adjust and respond to situations based on what they have learned. A descriptive phenomenological approach can identify commonalities in the lived experiences of people in similar situations with different contexts in relation to a common phenomenon [68]. Neubauer et al. [69] argues researchers addressing the subjective nature of a person’s thoughts have less reliable data than solid statistical evidence developed in a quantitative study. However, gaining emotionally rich information based on the lived experiences of people allows researchers to glean new insights into personal thoughts that would otherwise go unnoticed [70]. For this reason, a phenomenological approach was used for the study protocol because it provides an opportunity to contextualize individual experiences as a shared phenomenon [71] to uncover underlying social realities and expose institutional practices [72].

## 7. Study Design

The current qualitative study will use a descriptive phenomenological design [73] supported by Husserlian [74,75] philosophy. Phenomenology guides this inquiry to capture the lived experiences of participants within the context of a phenomenon [69], in this case caregiving, by probing their subjective consciousness [76]. The participants will share their experiences derived from the deep exploration of perceptions, feelings, memories, thoughts, and emotions [77]. The researchers will go directly to the source, the caregivers, to understand their lived experience managing the daily needs of a person living with a disease [39].

Because the experience is lived daily by the caregiver and evolves contextually with caring longevity, the caregiver can provide clarity and depth about the phenomenon to reveal deep hidden meanings [68]. Specifically, learning how caregivers cope with their stressors or manage physical fatigue can become the building blocks for developing strategies to support caregivers [64]. Support strategies are important since neurologists typically treat patients while ignoring the needs of caregivers [63]. For these reasons, a descriptive phenomenological study design is best suited to understand the lived experiences of women caring for their husbands living with PD.

## 8. Setting

Potential participants for the study will be recruited from the more than 500 members of the Colorado Parkinson Foundation. Most participants are anticipated to live in the El Paso County metropolitan area, which includes Colorado Springs. This area has a population of nearly 800,000. Colorado has about 11,500 people with PD [78] with an estimated diagnosis rate of 6.4 per 10,000 [79].

## 9. Sampling and Recruitment

### 9.1. Sampling

Participants will be recruited using purposive sampling [80], where participants are selected based on researcher judgment about the participants characteristics which will be most informative [81] for a deep analysis of the data [82]. The final sample size will be determined by data saturation [83], the point at which no new information is derived from the data [84]. Given the study design and narrow scope of the phenomenon, the number of participants is expected to range from 8 to 16 participants [85,86]. However, depending on participant homogeneity, as many as 30 participants may be necessary to achieve saturation [87].

### 9.2. Inclusion and Exclusion Criteria

Study participants will be mature females aged over 50 years who are the primary care caregiver of their male spouse living with PD classified between stages 2 to 4 with the Hoehn and Yahr Scale. These stages of PD progression were selected so potential participants would have experience with mild to severe disease stages; a higher numbered stage translates into more caregiver involvement with managing movement and ADLs. Stage 1 was excluded from the study because 70% of people living with PD are undiagnosed for five or more years [88]; most tend to mistake the mild symptoms with aging. Stage 5 was excluded because people living with PD at stage 5 are often moved to long-term care or nursing facilities; most tend to have full care requirements, the need for more medical services, or end-of-life issues [89]. A primary caregiver in the current study is a woman caring for their husbands for more than 40 h per week. Furthermore, the caregiving must be provided in the home rather than in an assisted living facility or nursing home. Finally, the caregiver needs to be able to verbally communicate in English.

### 9.3. Recruitment

**Figure 2:**
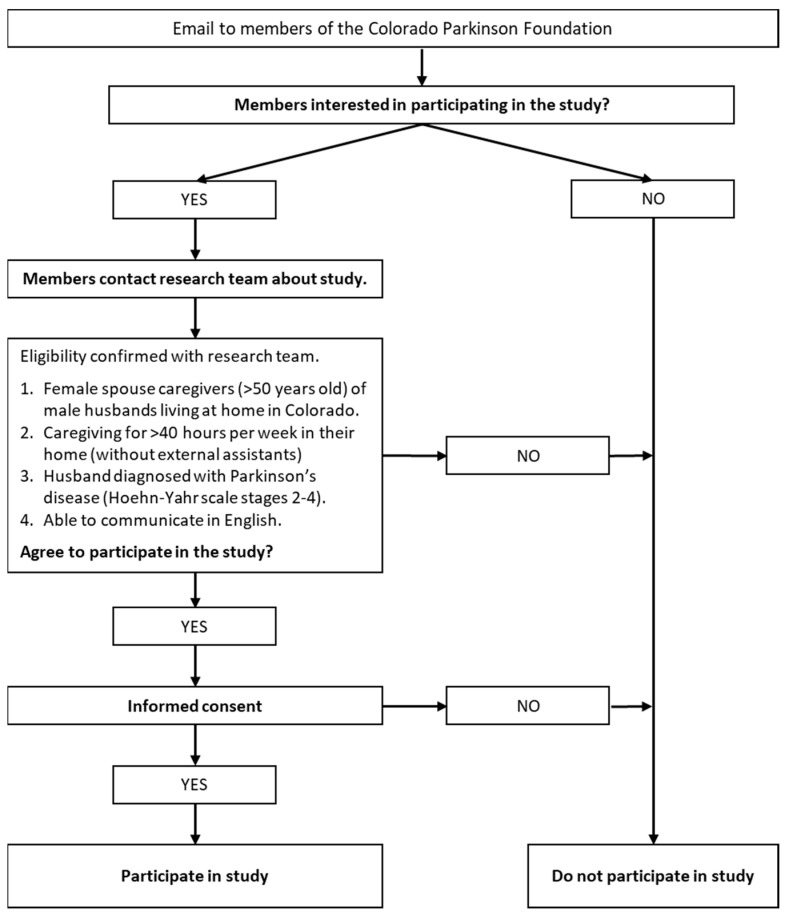
Recruitment Flowchart.

Women caregivers of husbands living with PD will be recruited from the membership of the Colorado Parkinson Foundation (Figure 2). To being the recruitment process, the study information, including contact information for the primary investigator, will be emailed to members. Announcements about the study will also be included in newsletters and provided at regularly scheduled member meetings. After contacting the primary investigator by email or telephone, the potential participant will be provided with more detailed information about the study. Next, the researcher will schedule an appointment with the potential participant to present an overview of the study guided by the informed consent information approved by the ethics committee. After the recruitment screening questions are answered, the primary investigator will ask potential participants about their willingness to participate in the study. If they agree to participate, the primary investigator will provide the participant with a link to SignUpGenius [90], an easy-to-use online program for scheduling appointments, so participants can schedule an interview at a convenient time.

## 10. Data Collection

The primary investigator will conduct the interviews, as recommended by Rubin and Rubin [91], to encourage the women to express themselves freely as they describe their caregiving experiences and identify their perceptions about caring for their husbands. The semi-structured interviews will be completed in a conversational style. This interview method provides flexibility for exploring the underlying reasons to describe the phenomenon [92]. The interviews are expected to be about 60 min. Due to COVID-19 restrictions and because women caregivers and their husbands are at higher risk for transmission, all interviews will be conducted face-to-face using Zoom© rather than in person. Zoom is an online videoconferencing extensively used for research [93,94,95,96,97,98].

The interview guide developed for the current study begins with 10 demographic questions and progresses to fourteen questions that explore three common areas referenced in the literature: caregiving, caregiver burden, caregiver benefit, and fatigue (Table 2). The interview guide is intended to facilitate a conversation with participants, where the researcher listens to participants describe their lived experiences using guides and prompts [99] rather than a directed [100] conversation. If new areas emerge during the conversation, the topics will be discussed with the research team and potentially incorporated into the continuing interview process [101]. The interviews will be conducted in a conversational manner, so participants feel comfortable responding to the questions with an accurate account of their lived experience.

More specifically, at the start of the interview, each participant will be asked to provide informed consent to participate in the study. Next, they will be asked for demographic information, including age, years of marriage, years of caregiving, hours per week of caregiving, information about work outside of the house, and duration of their husband’s disease. During the interviews, the primary investigator will make field notes to record contextual information about participant statements [103]. At the close of the interview, the participant will be asked to provide feedback about the major points noted by the interviewer. The completed interview will be sent to Rev [104] for transcription. Rev is a low-cost, high-quality transcription service that uses a secure online platform, and written transcripts will be produced verbatim by a professional health services transcriptionist within 24 h. This quick turnaround is important to begin the initial coding process. The primary investigator will compare each transcript with the audio recording to verify transcription accuracy [105]. The final transcripts will be uploaded into Atlas.ti (version 11) qualitative data analysis software for coding and thematic analysis.

## 11. Data Analysis

**Figure 3:**
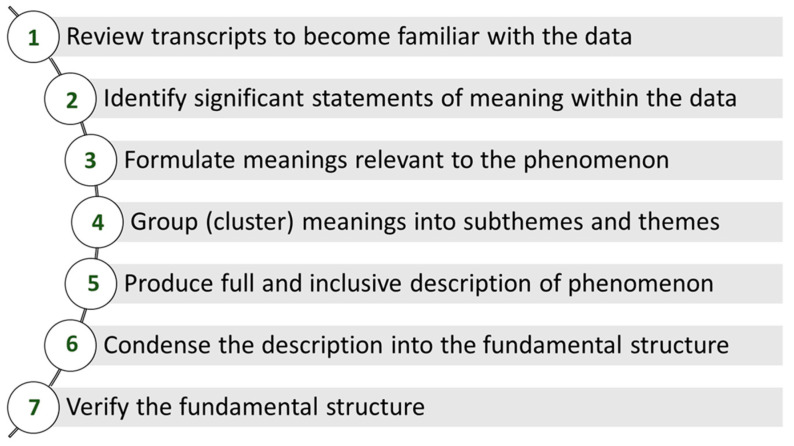
Colaizzi’s Analysis Process for Descriptive Phenomenology.

Data analysis will follow Colaizzi’s [106] seven-step process (Figure 3) to determine the fundamental structure and substantial themes that describe the lived experience of the participants. Because this data analysis process is designed for descriptive phenomenological studies, it aligns with our study design [107]. The seven steps of the process include the following: (1) creating familiarization with the data, (2) identifying significant statements of meaning, (3) formulating meanings, (4) clustering themes, (5) developing an exhaustive description, (6) producing the fundamental structure, and (7) seeking verification of the fundamental structure [108]. An important difference between this analytic process and the process proposed by Giorgi [109] is that the final structure is verified through member checking. From Giorgi’s [110] perspective, this step is incompatible with the method because the researcher and participant have differing “natural attitudes” about the phenomenon. However, participants should be able to recognize their lived experience as a caregiver from the fundamental structure produced by this study [108]. As such, this step serves an important role for validation of the fundamental structure from the perspective of the participants [111]. Through this process, descriptive phenomenology is able to elicit an exhaustive description about the phenomenon of caregiving [112].

In the current study, data analysis will begin immediately after transcription of the interviews. Additionally, each transcript will be compared with the audio recording by the primary investigator before the next scheduled interview. Throughout the data collection process, each transcript will be reviewed multiple times by the primary and secondary investigators to gain a deeper understanding of the content. Transcripts will also be shared with all research team members during data analysis. Specific statements determined to have a significant meaning will be extracted from the transcripts and scrutinized to determine deeper meanings. Statements will be organized by subthemes and then categorized into themes. Next, the themes will be organized to provide a coherent description of the lived experiences. From this data, the structure of the caregiver lived experiences will be summarized. Finally, the results will be sent to participants for feedback and verification.

## 12. Trustworthiness and Rigor

Trustworthiness of research data is a strategy used to ensure credibility, transferability, dependability, and confirmability [113,114]. Establishing trustworthiness requires researchers to construct a rigorous method with clearly stated procedures and to check the findings for accuracy [115]. Thus, credibility means the analysis is believable, transferability implies the information is applicable to another context, dependability indicates the findings can be reproduced, and confirmability means the results are supported by data [116]. Furthermore, the sampling strategy provides coherence through epistemological congruency with the study design [117]. Data in the current study will be analyzed independently by two researchers to ensure credibility and confirmability [114]. Methodological strategies will also be implemented to ensure the trustworthiness of the data, including an audit trail, bracketing, coding checks, categorization, constant feedback, research team meetings, peer debriefing with an external researcher, triangulation with other sources, and member checking [118,119,120,121]. By embedding these strategies in this rigorous process, the researchers will be able to establish the validity of the findings with credibility, authenticity, criticality, and integrity [122].

In addition, the primary investigator will make field notes during the interview. These notes will include important verbal content and nonverbal communication to inform the study data [103,105]. The research team will also engage in reflexive journaling, and an audit trail will be maintained for documenting decisions to ensure credibility and dependability of the study findings [123]. Reflexive journaling will take place before and after each interview session and after data coding to ensure that no biases are introduced into the study findings. Data collection will continue until the point of data saturation, which will be defined as the point at which no additional data is found that pertains to the phenomenon [85]. Audio recording and a completed professional transcription within 24 to 48 h after the interview will enhance rigor. The independent coding and auditing processes are strategies that enhance the confirmability of the results.

### Reflexive Bracketing

According to Chan et al. [124], Etherington [125], Gearing [126], and Weatherford and Maitra [127], prior knowledge, perspectives, or beliefs can influence data collection and result in a biased analysis. For this reason, the Husserlian approach for descriptive phenomenology [75] is characterized by the epoché or the “pure mode of apperception” [128]. In this approach, the researcher brackets, or holds, all preconceptions in abeyance when collecting and analyzing data [129]. By suspending preconceived notions and beliefs about the phenomenon, the data has more rigor [130], and the data collection and analysis processes are more reflective and introspective. Bracketing with the epoché is an important difference from Heideggerian interpretative phenomenology [131], where researcher assumptions shape the data collection process and impose understanding on constructed findings during data analysis [132]. In the current study, bracketing will begin with the research team acknowledging potential influences that could intercept or interrupt the analysis [133]. For the best results, the researchers need to be curious and quizzical, self-critical and self-aware, open and transparent, precise and insightful, and willing to be wrong [134]. Importantly, this process requires team communication [135] and mindfulness [136] to enhance reflective awareness and critical thinking. As the research team engages in reflective bracketing, the relevant meaning can emerge unobstructed from the data rather than constructed from the beliefs of the researchers.

## 13. Ethical Considerations

### 13.1. Contact

Potential participants will receive an emailed invitation letter from the research team through the Colorado Parkinson Foundation. The invitation letter will include a brief statement about the study aims and the participant’s involvement in the study. The contact information for the primary investigator will be provided in the letter. Potential participants interested in the study can directly contact the primary investigator to learn more about the study. Then, the primary investigator will meet with potential participants by telephone or Zoom© to explain the study using the approved information document from the local institutional review board. The total time required for participant enrollment will be no more than 2 h and 30 min, including 1 h for the email communication and participant review of the informed consent, 1 h for the audio recorded interview, and 30 min for member checking for the data analysis. A completed informed consent form will be required from the potential participant if they agree to participate in the study.

### 13.2. Consent

Because of the current COVID-19 pandemic, participants will be unable to physically sign the informed consent document in the presence of the primary investigator. Since study participants are older adults, they may have limited knowledge about electronically signing documents or attaching documents to emails. Therefore, potential participants will have a copy of the informed consent document sent to their preferred email account. They will be asked to read the consent document and to send a reply email message back to the primary investigator indicating whether they agree or decline to participate. Potential participants will have a week to decide about participation in the study. If no response is received, on days 3 and 7, the primary investigator will send an email reminder to ask potential participants whether they have additional questions before deciding to participate. After day 7, if no reply is received from the potential participant, they will be removed from the contact list. After they agree to participate in the study, participants will be asked to schedule their interview through SignUpGenius. Before beginning the interview in Zoom©, participants will again be asked whether they have any questions pertaining to informed consent and to verbally confirm that they agree to participate in the study. At this time, participants will be reminded that they have the right to withdraw from the study at any time and that participation is voluntary. In addition to electronic consent, this verbal consent will become part of the interview transcript.

### 13.3. Data Security and Confidentiality

To protect their confidentiality, study participants will receive a pseudonym from a list of flower names as their study identification for the duration of the study. The primary investigator will be responsible for data security and confidentiality. The transcription service complies with federal regulations for handling protected personal health information and provides a written confidentiality agreement. The participant demographic information will be gathered and stored separately from the deidentified information and will be deidentified from the final transcripts before data analysis. The researcher team will only work with deidentified participant data during the study. All digital data will be stored on password-protected computers, and deidentified transcripts will be stored in a locked file cabinet. All investigators have password-protected computers, with antivirus software and antispyware, for management of the data.

### 13.4. Digital Data Management

All paper documents, including field notes, will be uploaded into the primary investigator’s password-protected computer, and the paper document will be stored in secured file cabinet for 5 years. For the coding process, the audio recordings will be sent through a secure portal to the transcription service. The completed transcript will then be downloaded to the primary investigator’s computer for upload into Atlas.ti (version 11). Only deidentified data will be shared between the researchers assisting in data analysis.

### 13.5. Participant and Researcher Risks

There is minimal risk for participants since the interviews will be conducted with Zoom© at a location selected by the participant, and the data will be deidentified before analysis. Although unlikely, there is the possibility for psychological risk to participants caused by anxiety and stress as they speak about sensitive topics [137], such as their husband’s condition and their uncertain future. Additional psychological stressors can include worrying about future financial problems associated with the expense of caring for their spouse. For this reason, the participants will be informed that they can pause or stop the interview at any time without penalty. During the interview, the primary investigator will observe participants for signs of emotional distress or increasing anxiety [138]. Researchers are also at risk for emotional distress [139] when investigating sensitive topics [140] since they are immersed in the lived experiences of participants during the interview [141]. For this reason, the research team will observe each other for signs of emotional distress and address this possibility during every team meeting. The primary investigator conducting the interviews will also engage in reflection with another member of the research team to monitor for emotional distress.

## 14. Discussion

Women caring for their husbands living with PD face various challenges related to health, emotional, and financial concerns, yet they continue to act as the primary health advocate and caregiver. Their role as a caregiver is essential for maintaining ADLs for their husbands, attending medical appointments, and refilling medications. Wives also support and encourage their husbands as they cope with the progression of the disease. Because these caregivers primarily focus on aiding their husbands, they often neglect caring for themselves, leading to adverse health outcomes like lack of sleep, anxiety, and depression [28,42]. For this reason, the current study is important to advance our knowledge about the lived experiences of caregivers as they specifically relate to burdens and benefits. Because of the phenomenological design, the in-depth interviews will provide the context and content of the lived experience from the perspective of the participant.

## 15. Limitations

Since the study’s research protocol intends to recruit participants only from Colorado, the findings will not be generalizable to other populations or other locations. However, the findings will be specific to women caring for their husbands living with PD and should address the study aims of exploring caregiving, burdens, and benefits of these women. Given the sparse evidence about caregiver burden and benefit, this information may be particularly useful.

Another potential limitation is that participants who lack the technological ability to use email or Zoom© may not be self-excluded from participation. As such, findings may be biased because only women capable of managing technology agree to participate. Because of the COVID-19 pandemic, this limitation is difficult to address. However, the study protocol allows potential participants to request assistance from the Colorado Parkinson Foundation for assistance with the Zoom© interview. This option will be provided to potential participants with technology hesitancies to increase the opportunity for maximum participation.

Finally, interviews conducted with videoconferencing or telecommunication technologies rather than in-person face-to-face interviews should be evaluated for potential limitations during the data collection process [94,142,143,144,145]. Recent studies report good data collection experiences, including higher participant satisfaction, with face-to-face Zoom© interviews [93,95,98] when there are not technical difficulties [93]. For this reason, research teams need to carefully select a viable technology for their study design [96], participants need to be prepared to use technology for interviews, and the interviewers need to be ready to adjust to technological problems outside their direct control [93]. Any technological problems during the interviews need to be reported and potential bias introduced by technologies need to be identified as a limitation.

## 16. Results

The study design was developed according to international recommendations for reporting qualitative research. Specifically, the quality criteria recommended by the Standards for Reporting Qualitative Research (SRQR) [146] guided development of the protocol, and the Consolidated Criteria for Reporting Qualitative Research (COREQ) [147] guided the development of the interview process. These two instruments are complementary with multiple overlapping criteria, but COREQ is more focused on research team characteristics, reflectivity, data collection, and data reporting [148]. By using SRQR and COREQ, findings of the study will be reported with a “thorough, transparent, and trustworthy account of the data collection process, analysis, and the relationship to the findings” [149]. A completed consolidated checklist for the recommended reporting criteria will be included with the study findings as a supplementary table (see Table S1).

## 17. Conclusions

Few studies describe the lived experience of wives caring for their husbands living with PD. Most of the existing evidence for caregivers of people living with PD is derived from cross-sectional instrument studies. Therefore, deeper inquiries are needed regarding the phenomenon of caregiving in relation to burdens and benefits when the caregiver is a spouse caring for her husband. Development of the qualitative interview questions that will be used in the current study was informed by a comprehensive literature review focused on caregiving and PD disease to address gaps in the knowledge. Because findings from this study will likely contribute new knowledge to the existing literature, the current article provided an in-depth description of the study protocol for this descriptive phenomenological study. We believe findings from the current study may guide the development of strategies that increase the ability of clinicians to support caregivers, increase the services provided by other health care professionals, and support the caregiving process in the home. Finally, findings can result in recommendations for policy makers to support family caregivers through public institutions and initiatives.

## Figures and Tables

**Table 2:** Semi-Structured Interview Guide with Evidence Sources.

Interview Question	Evidence Sources	Construct(s)
Tell me about your experiences of caring for your husband with Parkinson’s disease.	Opening the conversation	Grand question
Please describe your experiences working with the physicians to manage the Parkinson’s disease physicians. How have they helped or hindered you? (Prompt: Are you addressed during visits? What can the physicians do to help you?)	Boersma et al., 2017 [23] Dekawaty et al., 2019 [64]	Wife caregiver Caregiver burden Caregiver benefit
What was your experience when your husband was first diagnosed with Parkinson’s disease? (Prompt: How did you feel? What did you think?)	Hoogland et al., 2019 [5] Schwartz et al., 2020 [63]	Wife caregiver
What disease symptoms are most challenging for you to manage? How do you deal with them? (Prompt: What might make these easier for you to manage?)	Boersma et al., 2017 [23] Smith et al., 2019 [12]	Caregiver burden Fatigue
Where do you find your strength? Please describe your inner strength. (Prompt: What helps you continue caring?)	Boersma et al., 2017 [23]	Caregiving Wife caregiver
Please describe what you normally do during the time you are caregiving. (Prompt: What does caring mean in the context of your work with your husband?)	Bakof et al., 2021 [36] Kang et al., 2020 [35] Theed et al., 2017 [26]	Caregiving Fatigue Wife caregiver
What is it like to go from being a wife and spouse to a full-time caregiver for a person living with Parkinson’s disease?	Balash et al., 2019 [11]	Caregiving Caregiver burden
Please describe your experience related to the progression of your husband’s Parkinson’s disease. (Prompt: What has been difficult?)	Juneja et al., 2020 [6] Smith et al., 2019 [12]	Caregiving Wife caregiver
As a caregiver for a person living with Parkinson’s disease, describe how your life has changed. (Prompt: Good, not so good, bad?)	Smith & Shaw, 2017 [32]	Caregiver burden Caregiver benefit
What is your experience with outside support you have received from the community, agencies, friends, and/or health providers?	Dekawaty et al., 2019 [64] Turney & Kushner, 2017 [31] Walga, 2019 [33]	Caregiving Caregiver burden
What are your experiences with adapting your life to your spouse’s Parkinson’s disease?	Dekawaty et al., 2019 [64] Hellqvist et al., 2020 [102]	Caregiving
In your own words, explain how you deal with the burden, or stress, of caring for your husband? (Prompt: Are there any benefits?)	Walga, 2019 [33]	Caregiver burden Caregiver benefit
Please explain in your own words what drives you to continue caring for your spouse? (Prompt: What are your motivators?)	APA, 2021 [40] Champagne & Muise, 2021 [34]	Caregiver benefit Wife caregiving
Is there anything else you would like to add?	Closing remarks	Final question

## Data Availability

Once the study is completed, data will be made available from the corresponding author on reasonable request.

## Supplementary Materials

The following supporting information can be downloaded at: https://www.mdpi.com/article/10.3390/jpm12050659/s1, Table S1: Consolidated Standard Criteria for Reporting Qualitative Research. References [146,147] are cited in the supplementary materials.
