# Supplementary material for: Women Caring for Husbands Living with Parkinson’s Disease: A Phenomenological Study Protocol
Source: J Pers Med. 2022 Apr 20;12(5):659. doi: 10.3390/jpm12050659 (PMC9146827; doi:10.3390/jpm12050659)
Supplement: Supplementary file 1 [file jpm-12-00659-s001.zip › jpm-1625828-supplementary.pdf]

Supplemental Table S1. Consolidated Standard Criteria for Reporting Qualitative Research

*Item No.	Section and Topic	Item with Description of the Criteria	***Page & Line #
**TITLE AND ABSTRACT**			
S-01	Title	Concise description of the nature and topic of the study. Identifying the study as qualitative or indicating the approach (e.g., ethnography, grounded theory) or data collection methods (e.g., interview, focus group) is recommended	
S-02	Abstract	Summary of key elements of the study using the abstract format of the intended publication; typically includes background, purpose, methods, results, and conclusions	
**INTRODUCTION / BACKGROUND**			
S-03	Problem formulation	Description and significance of the problem/phenomenon studied; review of relevant theory and empirical work; problem statement	
S-04	Purpose or research question	Purpose of the study and specific objectives or questions	
**METHODS**			
	**Study Design**		
S-05	Qualitative approach and research paradigm	Qualitative approach (e.g., ethnography, grounded theory, case study, phenomenology, narrative research) and guiding theory if appropriate; identifying the research paradigm (e.g., postpositivist, constructivist/interpretivist) is also recommended; rationale**	
C-09	Methodological orientation and Theory	What methodological orientation was stated to underpin the study? e.g. grounded theory, discourse analysis, ethnography, phenomenology, content analysis	
	**Setting**		
S-07	Context	Setting/site and salient contextual factors; rationale**	
	**Sampling**		
S-08	Sampling strategy	How and why research participants, documents, or events were selected; criteria for deciding when no further sampling was necessary (e.g., sampling saturation); rationale**	
C-10	Sampling	How were participants selected? *e.g. purposive, convenience, consecutive, snowball*	
C-11	Method of approach	How were participants approached? *e.g. face-to-face, telephone, mail, email*	
	**Data Collection**		
	Interviewer(s) Characteristics		

C-01	Interviewer/facilitator	Which author/s conducted the interview or focus group?	
C-02	Credentials	What were the researcher's credentials? E.g. PhD, MD	
C-03	Occupation	What was their occupation at the time of the study?	
C-04	Gender	Was the researcher male or female?	
C-05	Experience and training	What experience or training did the researcher have?	
	Research Team Characteristics		
S-06	Researcher characteristics and reflexivity	Researchers’ characteristics that may influence the research, including personal attributes, qualifications/experience, relationship with participants, assumptions, and/or presuppositions; potential or actual interaction between researchers’ characteristics and the research questions, approach, methods, results, and/or transferability.	
C-06	Relationship established	Was a relationship established prior to study commencement?	
C-07	Participant knowledge of the interviewer	What did the participants know about the researcher? e.g. personal goals, reasons for doing the research	
C-08	Interviewer characteristics	What characteristics were reported about the interviewer/facilitator? e.g. Bias, assumptions, reasons, and interests in the research topic	
	Setting		
C-14	Setting of data collection	Where was the data collected? e.g. home, clinic, workplace	
C-15	Presence of non-participants	Was anyone else present besides the participants and researchers?	
	Procedures		
S-10	Data collection methods	Types of data collected; details of data collection procedures including (as appropriate) start and stop dates of data collection and analysis, iterative process, triangulation of sources/methods, and modification of procedures in response to evolving study findings; rationale**	
S-11	Data collection instruments and technologies	Description of instruments (e.g., interview guides, questionnaires) and devices (e.g., audio recorders) used for data collection, if/how the instrument(s) changed over the course of the study	
C-17	Interview guide	Were questions, prompts, guides provided by the authors? Was it pilot tested?	
C-19	Audio/visual recording	Did the research use audio or visual recording to collect the data?	
C-20	Field notes	Were field notes made during and/or after the interview or focus group?	
C-21	Duration	What was the duration of the interviews or focus group?	
C-18	Repeat interviews	Were repeat interviews carried out? If yes, how many?	
	Data Management		

S-13	Data processing	Methods for processing data prior to and during analysis, including transcription, data entry, data management and security, verification of data integrity, data coding, and anonymization/deidentification of excerpts	
C-27	Software	What software, if applicable, was used to manage the data?	
C-22	Data saturation	Was data saturation discussed?	
	**Data Analysis**		
S-14	Data analysis	Process by which inferences, themes, etc., were identified and developed, including the researchers involved in data analysis; usually references a specific paradigm or approach; rationale**	
C-24	Number of data coders	How many data coders coded the data?	
C-25	Description of the coding tree	Did authors provide a description of the coding tree?	
C-26	Derivation of themes	Were themes identified in advance or derived from the data?	
	**Trustworthiness**		
S-15	Techniques to enhance trustworthiness	Techniques to enhance trustworthiness and credibility of data analysis (e.g., member checking, audit trail, triangulation); rationale**	
C-28	Participant checking	Did participants provide feedback on the findings?	
C-23	Transcripts returned	Were transcripts returned to participants for comment and/or correction?	
	**Ethical Considerations**		
S-09	Ethical issues pertaining to human subjects	Documentation of approval by an appropriate ethics review board and participant consent, or explanation for lack thereof; other confidentiality and data security issues	
S-20	Conflicts of interest	Potential sources of influence or perceived influence on study conduct	
**RESULTS / FINDINGS**			
	**Demographics**		
S-12	Units of study	Number and relevant characteristics of participants, documents, or events included in the study; level of participation (could be reported in results)	
C-12	Sample size	How many participants were in the study?	
C-16	Description of sample	What are the important characteristics of the sample? *e.g. demographic data, date*	
C-13	Non-participation	How many people refused to participate or dropped out? Reasons?	
	**Themes (Categories)**		
S-16	Synthesis and interpretation	Main findings (e.g., interpretations, inferences, and themes); might include development of a theory or model, or integration with prior research or theory	
C-31	Clarity of major themes	Were major themes clearly presented in the findings?	

C-32	Clarity of minor themes	Is there a description of diverse cases or discussion of minor themes?	
	**Data**		
S-17	Links to empirical data	Evidence (e.g., quotes, field notes, text excerpts, photographs) to substantiate analytic findings	
C-29	Quotations presented	Were participant quotations presented to illustrate the themes / findings? Was each quotation identified? *e.g. participant number*	
C-30	Data and findings consistent	Was there consistency between the data presented and the findings?	
**DISCUSSION**			
S-18	Integration with prior work, implications, transferability, and contribution(s) to the field	Short summary of main findings; explanation of how findings and conclusions connect to, support, elaborate on, or challenge conclusions of earlier scholarship; discussion of scope of application/ generalizability; identification of unique contribution(s) to scholarship in a discipline or field	
	**Study Limitations**		
S-19	Limitations	Trustworthiness and limitations of findings	
**CONCLUSIONS**			
New	Major findings summarized	A short summary about the important evidence presented in the findings; interpretation about the meaning of the analyzed data.	
New	Implication of the findings	Concise statements about the implication of the findings; how should the findings guide practice, policy, and/or research?	
**DISCLOSURES**			
S-20	Conflicts of interest	Potential sources of influence or perceived influence on study conduct	
S-21	Funding	Sources of funding and other support; role of funders in data collection, interpretation, and reporting	

* The item numbers indicate the checklist with the first letter (C for COREQ and S for SRQR) followed by the original item number.

** The rationale should briefly discuss the justification for choosing that theory, approach, method, or technique rather than other options available, the assumptions and limitations implicit in those choices, and how those choices influence study conclusions and transferability. As appropriate, the rationale for several items might be discussed together (Note from, the SRQR).

*** p: present; np: not present; n/a: not applicable (Note from the SRQR).

Supplemental Table Notes

– The Consolidated Standards for Reporting Qualitative Research integrates the Standards for Reporting Qualitative Research (SRQR) 21-item checklist, and the Consolidated Criteria for Reporting Qualitative Studies (COREQ) 32-item checklist with an additional 2-items (new) in order of the normal sections of a qualitative research manuscript – Introduction/Background, Methods, Results/Findings, Conclusions, and Disclosures.

References

- O'Brien, B.C.; Harris, I.B.; Beckman, T.J.; Reed, D.A.; Cook, D.A. Standards for reporting qualitative research: A synthesis of recommendations. Acad. Med. **2014**, 89, 1245–1251. https://doi.org/10.1097/acm.0000000000000388.
- Tong, A.; Sainsbury, P.; Craig, J. Consolidated criteria for reporting qualitative research (COREQ): A 32-item checklist for interviews and focus groups. Int. J. Qual. Health Care **2007**, 19, 349–357. https://doi.org/10.1093/intqhc/mzm042.
